# High-plex protein and whole transcriptome co-mapping at cellular resolution with spatial CITE-seq

**DOI:** 10.1038/s41587-023-01676-0

**Published:** 2023-02-23

**Authors:** Yang Liu, Marcello DiStasio, Graham Su, Hiromitsu Asashima, Archibald Enninful, Xiaoyu Qin, Yanxiang Deng, Jungmin Nam, Fu Gao, Pino Bordignon, Marco Cassano, Mary Tomayko, Mina Xu, Stephanie Halene, Joseph E. Craft, David Hafler, Rong Fan

**Affiliations:** 1 Department of Biomedical Engineering, Yale University, New Haven, CT USA; 2 Yale Stem Cell Center and Yale Cancer Center, Yale School of Medicine, New Haven, CT USA; 3 Department of Pathology, Yale School of Medicine, New Haven, CT USA; 4 Department of Medicine, Yale School of Medicine, New Haven, CT USA; 5 Department of Neurology, Yale School of Medicine, New Haven, CT USA; 6 Lunaphore Technologies SA, Tolochenaz, Switzerland; 7 Department of Dermatology, Yale School of Medicine, New Haven, CT USA; 8 Department of Immunobiology, Yale School of Medicine, New Haven, CT USA; 9 Human and Translational Immunology Program, Yale School of Medicine, New Haven, CT USA

**Keywords:** Proteomics, Gene expression analysis, Gene expression profiling

## Abstract

In this study, we extended co-indexing of transcriptomes and epitopes (CITE) to the spatial dimension and demonstrated high-plex protein and whole transcriptome co-mapping. We profiled 189 proteins and whole transcriptome in multiple mouse tissue types with spatial CITE sequencing and then further applied the method to measure 273 proteins and transcriptome in human tissues, revealing spatially distinct germinal center reactions in tonsil and early immune activation in skin at the Coronavirus Disease 2019 mRNA vaccine injection site.

## Abstract (web summary)

Co-indexing of transcriptomes and epitopes is extended to the spatial dimension with large protein panels.

## Main

Spatially resolved transcriptome sequencing has generated biological insights in the study of cell differentiation and tissue development^1–3^ but does not yet incorporate measurements of large protein panels. Previously, we developed microfluidic deterministic barcoding in tissue (DBiT) for co-mapping of whole transcriptome and a panel of 22 proteins at the cellular level (~10-µm pixel size) using antibody-derived DNA tags (ADTs)^4^ to convert the detection of proteins to the sequencing of corresponding DNA tags^5,6^. Array-based spatial transcriptome was also expanded to multi-omics, namely SM-Omics^7^, which demonstrated the mapping of six proteins and whole transcriptome with 100-µm spot size. Very recently, Landau et al.^8^ further implemented spatial multi-omics on the 10x Visium platform with 55-µm spot size and a panel of 21 protein markers. Spatial proteogenomic profiling of liver tissue demonstrated highly multiplexed (~100) protein measurement using Visium^9^. However, it remains unclear how large a panel of proteins can be simultaneously mapped and what difference can be obtained if high-plex (>100) protein mapping was realized.

**Fig. 1:**
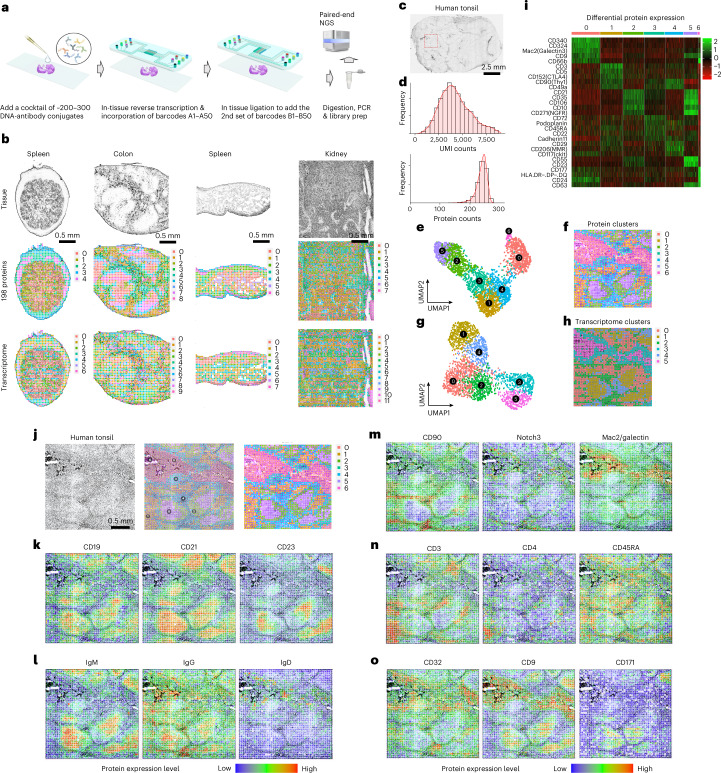
Spatial-CITE-seq workflow design and application to diverse mouse tissue types and human tonsil for co-mapping of proteins and whole transcriptome. **a**, Scheme of spatial-CITE-seq. A cocktail of ADTs is applied to a PFA-fixed tissue section to label a panel of ~200–300 protein markers in situ. Next, a set of DNA barcodes A1–A50 is flowed over the tissue surface in a spatially defined manner via parallel microchannels, and reverse transcription is carried out inside each channel for in-tissue synthesis of cDNAs complementary to endogenous mRNAs and introduced ADTs. Then, a set of DNA barcodes B1–B50 is introduced using another microfluidic device with microchannels perpendicular to the first flow direction and subsequently ligated to barcodes A1–A50, creating a 2D grid of tissue pixels, each of which has a unique spatial address code AB. Finally, barcoded cDNA is collected, purified, amplified and prepared for paired-end NGS sequencing. **b**, Spatially resolved 189-plex protein and whole transcriptome co-mapping of mouse spleen, colon, intestine and kidney tissue with 25-µm pixel size. Upper row: bright-field optical images of the tissue sections. Middle row: unsupervised clustering of all pixels based on all 189 protein markers only and projection onto the tissue images. Lower row: unsupervised clustering of whole transcriptome of all pixels and projection to the tissue images. Colors correspond to different proteomic or transcriptomic clusters indicated on the right side of each panel. **c**, Image of a human tonsil tissue section. The region mapped by spatial-CITE-seq is indicated by a dashed box. **d**, Per-pixel UMI count and protein count histograms. **e**, UMAP plot of the clustering analysis of all pixels based on 273 proteins only. **f**, Spatial distribution of the clusters (0–6) indicated by the same colors as in **e**. **g**, UMAP plot of the clustering analysis of all pixels based on the mRNA transcriptome. **h**, Spatial distribution of the transcriptomic clusters (0–5) indicated by the same colors as in **g**. Pixel size: 25 µm. **i**, Differentially expressed proteins in the clusters shown in **c** and **d**. **j**, Tissue image of the mapped region (left), spatial proteomic clusters (right) and the overlay (middle). **k**, Individual surface protein markers related to B cells and follicular DCs. **l**, Functional protein markers such as immunoglobulins showing spatially distinct distribution of GC B cells (IgM), matured B cells (IgG) and naive B cells (IgD), in agreement with B cell maturation, class switch and migration. **m**, Individual protein markers enriched in the extracellular region (CD90, Notch3) and crypt (Mac2). **n**, Individual T cell protein markers CD3, CD4 and CD45RA showing T cell zones and subtypes. **o**, Individual protein markers CD32, CD9 and CD171. CD32 identified a range of immune cells, including platelets, neutrophils, macrophages and DCs, trafficking from vasculature. CD9 identified plasma cell precursors in GCs and crypt. CD171, a neural cell adhesion molecule, is found highly distinct in the GC dark zone. Color key: protein expression from high to low.

**Extended Data Fig. 1:**
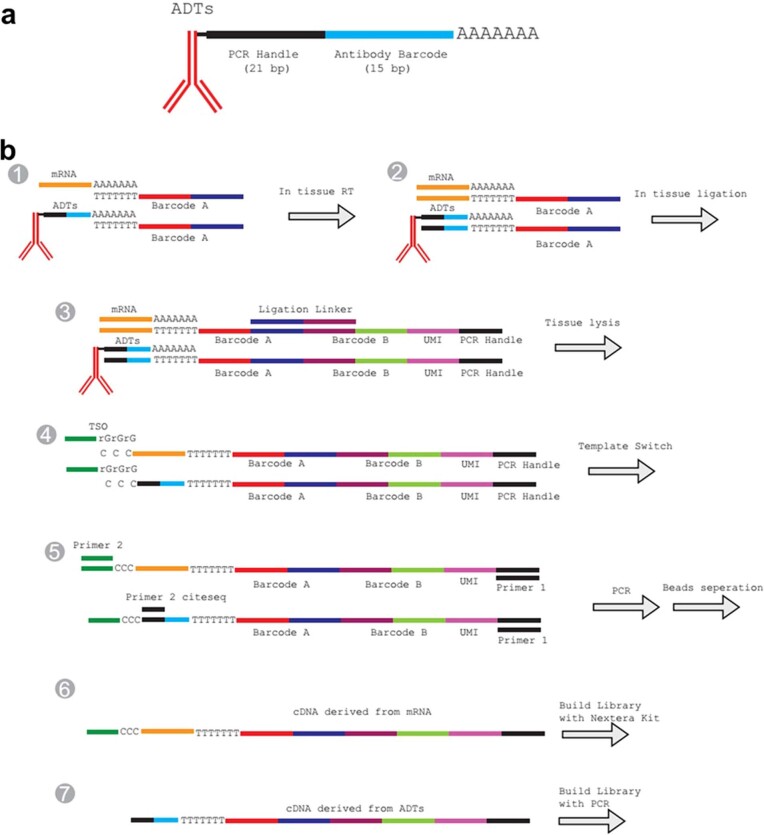
Spatial-CITE-seq design and detailed workflow. (**a**) ADT structure. The oligo labelled to the antibody has three functional regions: PCR handle (21 bp), antibody barcode (15 bp) and poly-A region (32 bp). (**b**) ADTs and mRNA with Poly-A region at the 3′ end can be reverse transcribed into cDNA using Barcode A as the RT primer. Barcode A consists of three functional regions, the poly-T region, spatial barcode region and the ligation region. During the first flow, 50 Barcode As were loaded into 50 parallel channels and the RT reaction was carried out inside each isolated channel (Step 1&2). After peeling off the 1st PDMS, a 2nd PDMS was attached. The in-channel ligation was performed with injecting 50 Barcode Bs into each of the 50 channels which are perpendicular to the channels of 1st PDMS chip (Step 3). Barcode B has four functional regions: ligation region, barcode region, UMI region and PCR handle region. Barcode B was also 5′ biotin modification. After ligation, tissue was lysed, and cDNAs were purified with streptavidin beads. The cDNAs on the beads were templated switched with template switch oligo (Step 4). PCR was used to amplify the cDNA (Step 5). The products were split into two portions, the mRNA derived cDNAs and the ADT derived cDNAs. The library was then built separately. More details were in the method section.

Here we report on spatial co-indexing of transcriptomes and epitopes for multi-omics mapping by highly parallel sequencing (spatial-CITE-seq), which uses a cocktail of ~200–300 ADTs to stain a tissue slide, followed by deterministic in-tissue barcoding of both DNA tags and mRNAs for spatially resolved high-plex protein and transcriptome co-profiling (Fig. 1a). Each ADT contains a poly(A) tail, a unique molecular identifier (UMI) and a specific DNA sequence unique to the corresponding antibody (Extended Data Fig. 1a). A large panel of ADTs was combined in a cocktail and applied to a paraformaldehyde (PFA)-fixed tissue section (~7 µm in thickness). Next, a microfluidic chip was used to introduce to the tissue surface a panel of DNA row barcodes A1–A50, each of which contains an oligo-dT sequence that binds to the poly(A) tail of ADTs or mRNAs, followed by in-tissue reverse transcription. Then, a panel of DNA column barcodes B1–B50 was flowed over the tissue surface in a perpendicular direction using a different microfluidic chip and ligated in situ to create a two-dimensional (2D) grid of tissue pixels, each containing a unique spatial address code AiBj (i = 1–50 and j = 1–50) to co-index all protein epitopes and transcriptome. Finally, barcoded cDNAs were recovered, purified and polymerase chain reaction (PCR) amplified to prepare two next-generation sequencing (NGS) libraries for paired-end sequencing of ADTs and mRNAs, respectively, for computational reconstruction of spatial protein or gene expression map.

**Extended Data Fig. 2:**
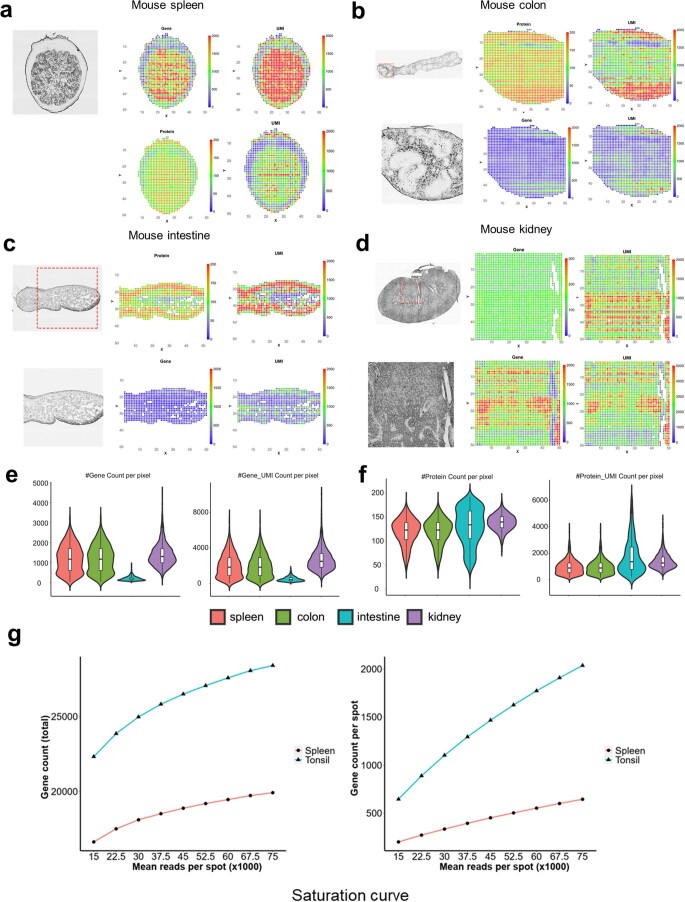
Spatial mapping of mouse spleen, colon, intestine and kidney with Spatial-CITE-seq. A 189 antibodies cocktail was used for all four mouse samples. The bright field image, spatial gene heatmap, spatial gene UMI heatmap, spatial protein heatmap and spatial protein UMI heatmap of spleen (**a**), colon (**b**), intestine (**c**) and kidney (**d**). (**e**) gene and gene UMI count per pixel of all four mouse samples. The box plots were derived from n = 2500 spatial pixels. The boxplot ranges from the first to the third quartile with the median value shown as the middle line, and whiskers represent 1.5× the interquartile range. (**f**) Protein and protein UMI count per pixel of all four mouse samples. (**g**) Transcriptome sequencing saturation curve of mouse spleen and human tonsil.

It was first demonstrated for spatial mapping of 189 proteins and genome-wide gene expression in multiple mouse tissue types, including spleen, colon, intestine and kidney. The mouse ADT panel (Supplementary Table 2) includes the markers for canonical cell types and immune cell function. The total number of proteins detected is approaching ~190, indictive of high sensitivity to detect even non-specific background noises. In the mouse spleen sample, the average protein count per pixel (25 µm) is 118, and the protein UMI account per pixel is 885 (Extended Data Table 1). Low UMI count pixels are localized in the low cell density capsule region (Extended Data Fig. 2). Uniquely, unlike our previous work that mapped much a smaller number of proteins and did not perform well on tissue region clustering analysis using the protein profiles alone, this high-plex protein panel allowed for unbiased clustering of all tissue pixels into spatially distinct clusters. Spatial protein profiles in the spleen sample resulted in five major clusters (Fig. 1b). Clusters 0 and 1 separate red and white pulps. Cluster 2 indicates microvascular tissue. Clusters 3 and 4 are enriched in spatially distinct regions of the capsule. Spatial transcriptome data from the same tissue section are of high quality (average gene count and UMI count per pixel: 1,166 and 1,972) (Extended Data Table 1). Transcriptome clustering analysis identified seven clusters that also resolved red and white pulps in concordance with spatial high-plex protein clustering. Mouse colon, intestine and kidney tissues were also analyzed, and the resultant major clusters correlated with anatomic regions (Fig. 1b).

We further conducted spatial co-mapping of 273 human protein markers (Supplementary Table 2) and whole transcriptome in human secondary lymphoid (tonsil) tissue over a 2.5 mm × 2.5 mm region of interest (indicated by a dashed box in Fig. 1c). Average protein count per pixel is 239, with the average UMI count of 4,309 (Fig. 1d and Extended Data Table 1). We also conducted the sequencing saturation analysis of mouse spleen and human tonsil and found that more genes can be recovered if using a deeper sequencing depth (Extended Data Fig. 2g). Clustering of spatial protein profiles alone identified seven major clusters (Fig. 1e), and the corresponding spatial distribution showed highly distinct features (Fig. 1f). Spatial transcriptome obtained in this experiment gave rise to eight major clusters (Fig. 1g), and their spatial distribution (Fig. 1h) correlated well with spatial protein clusters but appeared to be more noisy and less precise. Differential protein expression analysis (Fig. 1i) allowed for identification of major cell types in each cluster. Overlay of tissue image and spatial protein cluster map (Fig. 1j) showed a strong correlation between anatomic features and tissue/cell types. Cluster 0 corresponds to the crypt epithelia. Clusters 2 and 5 are the germinal center (GC) light and dark zones. Cluster 1 indicates specific T cell zones. Clusters 3 and 4 are localized in extrafollicular regions. Cluster 6 contains peripheral blood cells in vasculature. We further visualized individual proteins one by one. For example, CD19, a marker for B cells, is enriched in follicles^10^. CD21 or complement receptor 2 (CR2)^11^, present on all mature B cells as well as follicular dendritic cells (DCs), is highly expressed in the whole follicles. CD23, previously found on mature B cells, activated macrophages, eosinophils, follicular DCs and platelets, is restricted to the apical region of the GC light zone^12^. We further examined the functional proteins, such as immunoglobulins, associated with B cell differentiation and maturation (Fig. 1k). IgM expression is restricted to GC B cells. Once they further mature, these B cells start to produce IgG and migrate out of follicles. IgD is produced mainly by naive B cells that just exit from the bloodstream (Fig. 1l). CD90 (Thy-1) is associated with a wide range of cell types but completely absent in GCs. Notch3 is found in squamous epithelial cells. Mac2/Galectin3 is highly enriched in the crypt zone (Fig. 1m). We also examined T cell marker CD3 that identified all major T cell zones as well as CD4 for helper T cells and CD45A for naive or stem-cell-like T cells (Fig. 1n). CD32 is an Fc receptor that regulates B cell activation^13^ and was found mainly outside GCs. CD9 is expressed in tonsillar B cells in both follicles and crypts. CD171, a neuronal cell adhesion molecule implicated in neurite outgrowth, myelination and neuronal differentiation, is found to be highly restricted in the dark zone. To our knowledge, this has not been reported previously and warrants further investigation (Fig. 1o).

**Extended Data Fig. 3:**
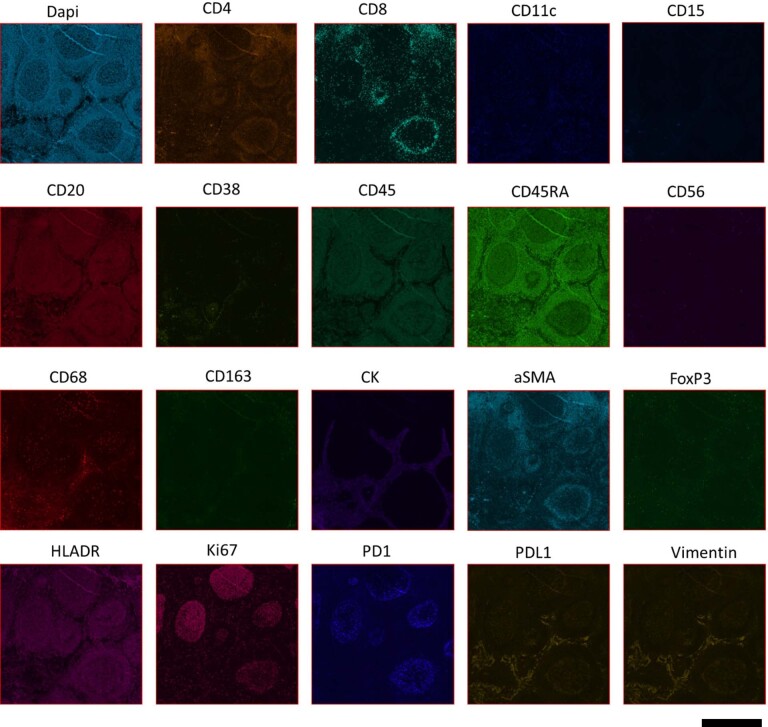
Immunostaining validation of spatial protein profiles. Sequential IF staining of human tonsil on COMET™ using the FFeX technology previously described by Lunaphore Technologies. Note: the data obtained is not from the same sample. Scale bar = 1 mm for all images. The experiment was from reference and was completed only once.

**Extended Data Fig. 4:**
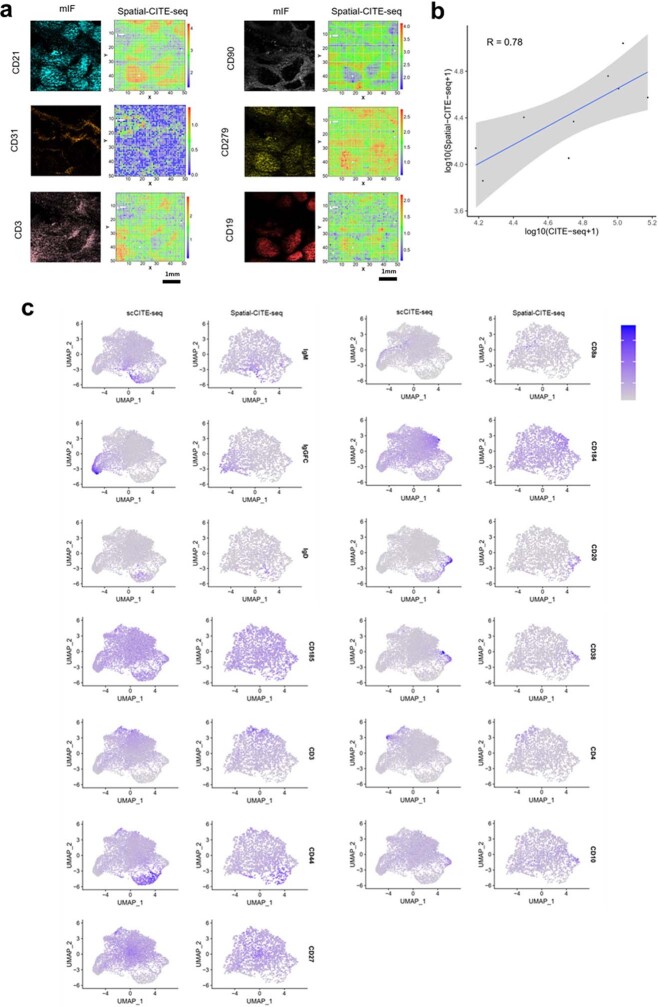
Comparison with single cell CITE-seq and immunofluorescence imaging (mIF). (**a**) Multiplex immunofluorescence imaging of 6 select proteins of an adjacent tissue section (human tonsil) and comparison with the protein expression map from spatial-CITE-seq. Color key: protein expression from high to low. The image was taken without repeats. (**b**) Person correlation analysis of pseudo bulk data generated from Spatial-CITE-seq and scCITE-seq data of human tonsil; The fitted linear regression line is in blue color and the 95% confidence interval was shown in gray color. (**c**) Integration analysis of Spatial-CITE-seq and scCITE-seq data from human tonsil.

**Extended Data Fig. 5:**
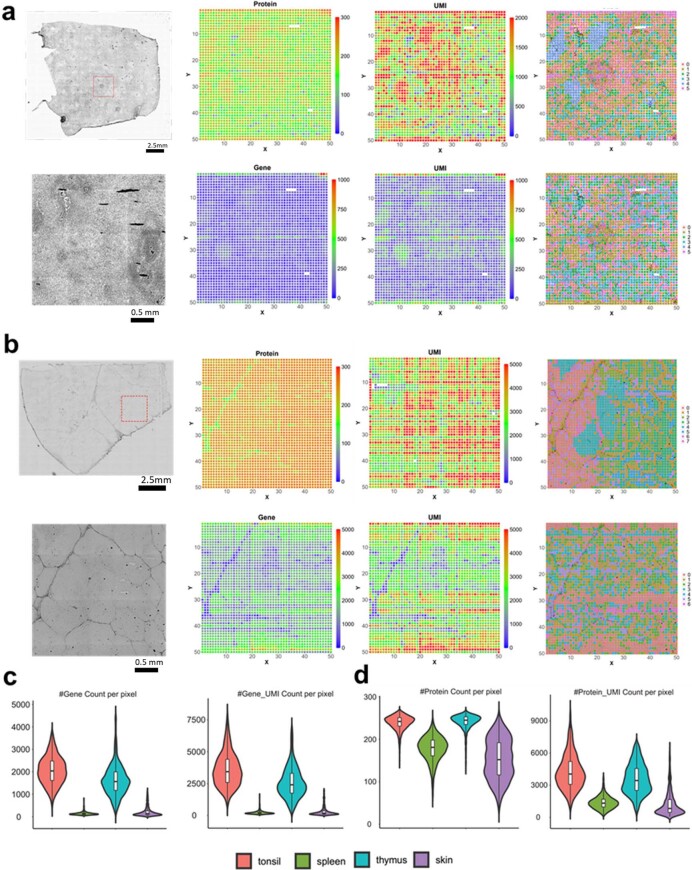
Spatial mapping of human spleen and thymus with Spatial-CITE-seq. A 273 antibodies cocktail was used for all four human samples. The bright field image, spatial gene heatmap, spatial gene UMI heatmap, spatial protein heatmap, spatial protein UMI heatmap, spatial clustering (based protein) and spatial clustering (based on RNA) of spleen (**a**) and thymus (**b**). (**c**) gene and gene UMI count per pixel of all four human samples. (**d**) Protein and protein UMI count per pixel of all four human samples. The box plots were derived from n = 2500 spatial pixels. The boxplot ranges from the first to the third quartile with the median value shown as the middle line, and whiskers represent 1.5× the interquartile range.

We conducted validation for selected proteins using multiplexed immunofluorescence imaging (Extended Data Figs. 3 and 4)^14^. In particular, using an adjacent tissue section, we conducted a head-to-head comparison for selected protein markers (Extended Data Fig. 4a). CD21, CD279 and CD19 were mainly detected within the GCs of tonsil. T cell markers CD90 and CD3 were observed mainly in the regions surrounding the GCs. CD31, an endothelial cell marker, depicts the vasculature, and its spatial pattern corresponds well to that obtained by spatial-CITE-seq. We next validated the spatial-CITE-seq by comparing it with single-cell CITE-seq (scCITE-seq). The pseudo-bulk data generated from spatial-CITE-seq were compared with those obtained from scCITE-seq data^15^, and a strong correlation was observed, with an R value of 0.78 (Extended Data Fig. 4b). We further integrated scCITE-seq and spatial-CITE-seq datasets using the Seurat integration package, which revealed that the two datasets share highly concordant protein expression patterns in 2D uniform manifold approximation and projection (UMAP) even for the low-frequency cell populations (Extended Data Fig. 4c). In addition, we also demonstrated the applicability of spatial-CITE-seq to other human tissues, including spleen and thymus (Extended Data Fig. 5).

**Fig. 2:**
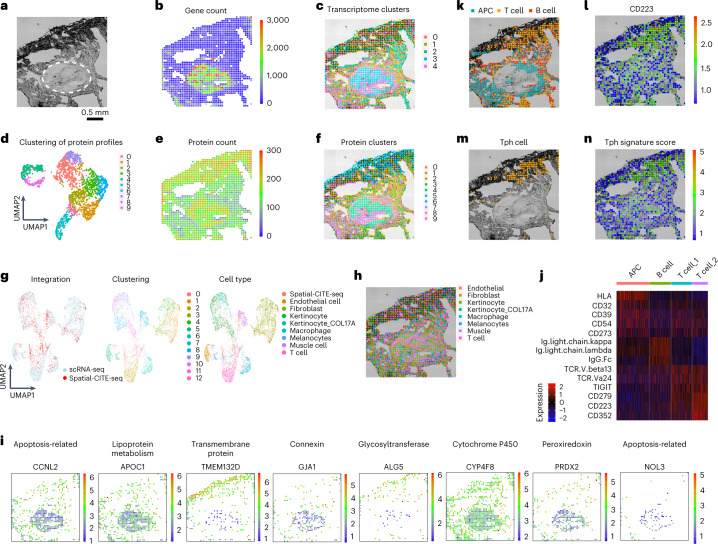
Integrated spatial and single-cell profiling of a human skin biopsy tissue at the site of COVID-19 mRNA vaccination injection revealed localized peripheral T cell activation. **a**, Bright-field image of skin section in the mapped region. A pilosebaceous unit is indicated by the dashed region. **b**, Gene count spatial map. **c**, Spatial clustering of all pixels based on whole transcriptome. Despite low gene count in the low cell density regions of dermal collagen, the clustering analysis revealed spatially distinct zones based on transcriptomic profiles. **d**, UMAP clustering of all 273 proteins. **e**, Protein count distribution. **f**, Spatial clustering of all pixels based on 273 proteins only, which is in high concordance with spatial clusters identified by spatial transcriptome co-mapped on the same tissue section. **g**, Integrated analysis of single-cell and spatial transcriptome. Left: The transcriptomes of spatial tissue pixels (red) conform to the clusters identified by joint analysis with scRNA-seq (blue). Middle: unsupervised clustering of the combined transcriptome dataset. Right: cell type annotation. **i**, Visualization of select genes associated with different gene oncology functions via integrated analysis and transfer learning. **j**, Differential protein expression in different cell types (APC, B cell and two subtypes of T cells). **k**, Spatial distribution of APCs, T cells and B cells. **l**, Expression of CD223 (LAG3) protein, a functional marker of activated T cells and other immune cell subsets. **m**, Identification of a highly localized population of Tph cells at the vaccine injection site. **n**, Spatial distribution of Tph gene score correlates with the cell localization. Pixel size: 25 µm.

**Extended Data Fig. 6:**
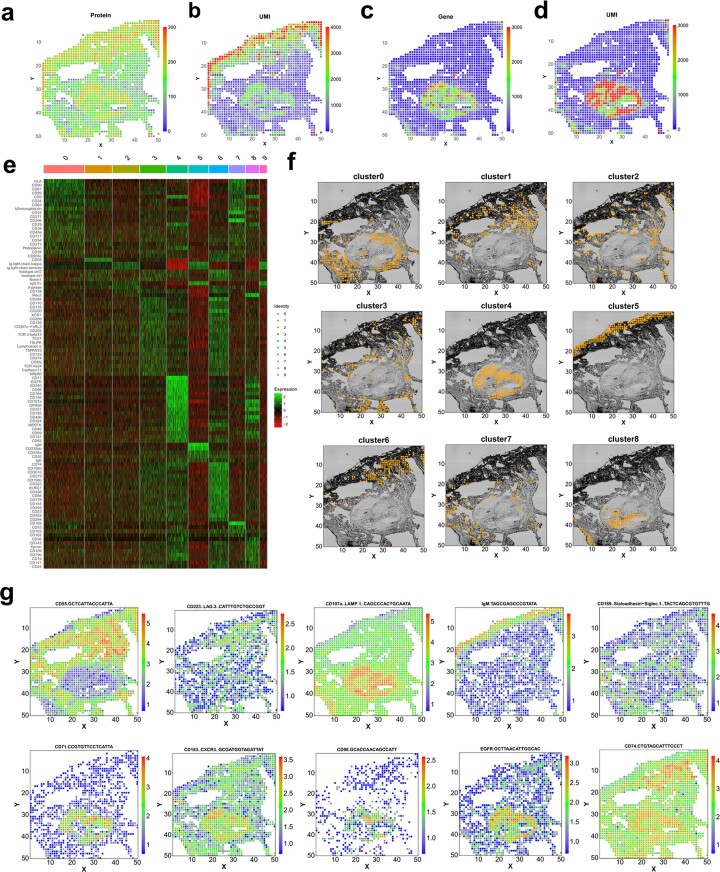
Spatial profiling of human skin biopsy tissue collected from the COVID-19 mRNA vaccine injection site. Spatial heatmap of gene (**a**), gene UMI (**b**), protein (**c**) and protein UMI (**d**). (**e**) Expression heatmap of the 10 clusters identified in skin biopsy sample. (**f**) the individual clusters plotted. (**g**) spatial distribution of some representative proteins.

**Extended Data Fig. 7:**
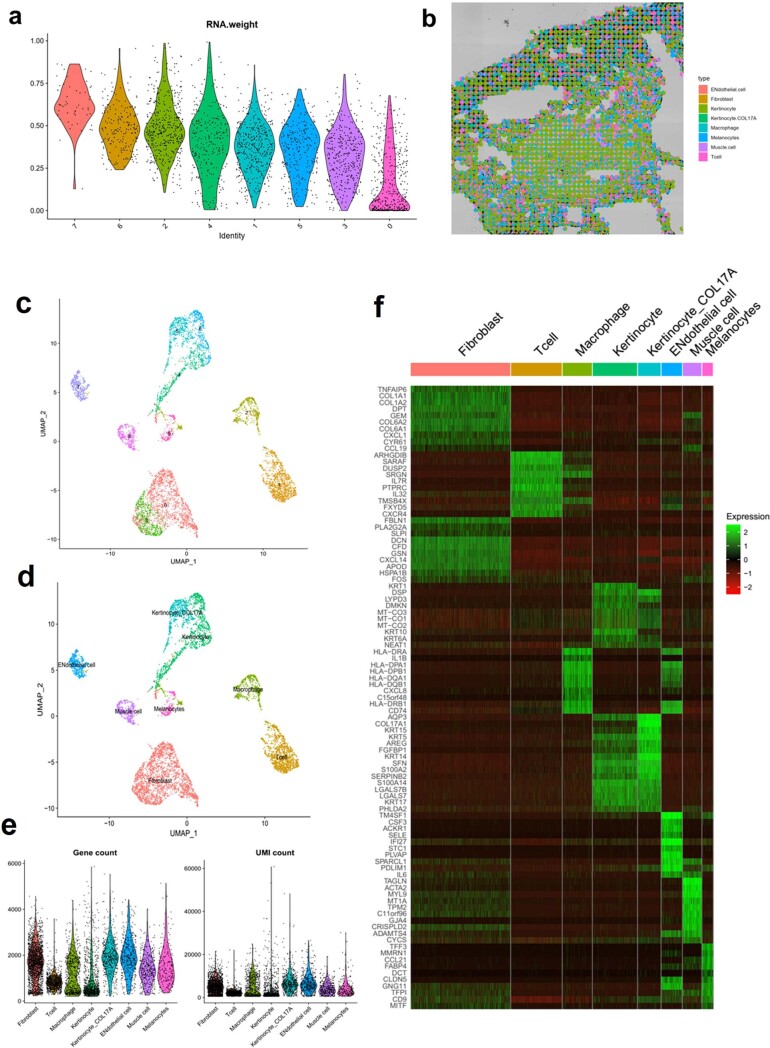
scRNA-seq sequencing data of skin biopsy sample and weighted-nearest neighbor analysis and deconvolution of Spatial CITE-seq data. (**a**) The modality weights that were learned for each cluster. Most of the clusters were weighed heavily on protein. (**b**) The spatial Pi chart generated using Spotlight package. The single cell reference was obtained from the same skin block. (**c**) spatial clusters of scRNA-seq data. (**d**) annotated cell types using canonical marker genes. (**e**) violin plot of genes and UMIs for each cell type. (**f**) Expression heatmap of different cell types.

Finally, spatial-CITE-seq was used to map early immune cell activation in a skin biopsy tissue collected from the Coronavirus Disease 2019 (COVID-19) mRNA vaccine injection site. The tissue section is comprised of collagen-rich region with low cell density and a vascular granule region with high cellularity (Fig. 2a). We evaluated the data quality for both transcriptome and proteins (Extended Data Fig. 6). Spatial map of gene count correlates with cell density, and the high cell density region resulted in 411 genes per pixel (Fig. 2b). However, unsupervised clustering identified spatially distinct clusters even in the low cell density regions (Fig. 2c). Spatial map of protein count is less variable across the tissue section, and up to ~270 proteins could be detected in the low density region (Fig. 2e). Clustering of spatial protein profiles gave rise to ten clusters (Fig. 2d), and the corresponding spatial distribution (Fig. 2f) was highly distinct in strong agreement with the spatial transcriptome clusters. Weighted nearest neighbor analysis was also conducted to identify the modality weight of RNA and protein in each of the spatial spots (Extended Data Fig. 7a).

Single-cell RNA sequencing (scRNA-seq) was conducted with the same skin biopsy tissue specimen (Extended Data Fig. 7). It was combined with spatial transcriptomes to perform clustering that gave rise to 13 major clusters, and the major cell types were identified based on gene oncology (Fig. 2g). Label transfer of cell types from scRNA-seq to spatial tissue pixels allowed for visualization of the distribution of different types (Fig. 2h). We can also visualize the expression of individual genes (Fig. 2i). For example, *CCNL2* and *NOL3*, which are apoptosis-related genes, were expressed in the vascular region; *APOC1* (responsible for lipoprotein metabolism), *GJA1* (connexin protein encoding) and *PRDX2* (peroxiredoxin encoding) were expressed mainly in the vascular. Transmembrane protein-encoding genes *TMEM132D* and glycosyltransferase *ALG5* were both expressed in the dermis region. *CYP4F8*, encoding CYP450 protein, was shown in most skin regions. The whole transcriptome sequencing could identify the cell types in general but were not specific enough here to show the different populations of T cells. Next, we focused on several immune cell types, including antigen-presenting cells (APCs), B cells and two subsets of T cells, as indicated by differentially expressed proteins (Fig. 2j). APCs and T cells are localized in spatially distinct regions, whereas B cells are distributed throughout the tissue (Fig. 2k). Specifically, T cell subset 2 expresses a set of markers, including lymphocyte activation gene 3 (LAG3)^16^, associated with peripheral helper T (Tph) cell population^17^ (Fig. 2m) as definitely by Tph signature score defined by expression levels of LAG3, PD-1 and CXCR6 (Fig. 2n). Tph cells are implicated in local T cell activation in response to vaccination. Thus, through integration of spatial high-plex protein and transcriptome mapping with scRNA-seq data from the same skin biopsy tissue, we identified major skin and immune cell types and a subset of Tph cells highly enriched at the injection site, which may contribute to the local immune activation that initiates systemic vaccine response. We also used the SPOTlight^18^ package to deconvolve the spatial spot and found that most cells were keratinocytes and fibroblasts, which matches the scRNA-seq data (Extended Data Fig. 7b).

Latest advances in imaging-based protein mapping, such as imaging mass cytometry (IMC)^19^ or multiplex immunofluorescence (that is, CODEX^14^, CyCIF^20,21^ and seqIF^22^), has realized 25–100-plex protein mapping and transformed spatial protein biomarker research. Our work used spatial barcoding and high-throughput sequencing for the mapping of ~200–300 proteins, representing the highest multiplexing to date for spatial protein profiling despite the lack of subcellular resolution. It could be expanded to >1,000-plex protein mapping given that only ~10% of the sequencing lane was used for the ADT library. We noticed a competition between ADTs and mRNAs for in-tissue reverse transcription and lower efficiency to detect transcripts compared to single-modality spatial transcriptome sequencing. This requires future optimization, such as ADT concentration and enzymatic reaction conditions. The current protein panel largely comprises surface epitopes and has yet to be further expanded to intracellular proteins or extracellular matrix proteins to investigate a wide range of protein signaling and function. In short, spatial-CITE-seq incorporates ~200–300 protein markers and offers substantial enhancement in the capabilities of tissue mapping, with applications to unmet needs in a wide range of fields, including cancer, immunology, infectious disease and anatomic pathology.

## Methods

### Microfluidic device design and fabrication

We designed the photomask using Autodesk AutoCAD 2021 and had the chrome mask printed by Front Range Photomasks with high resolution (2 µm). The chrome mask was cleaned extensively with acetone and air dried before use. Polymethylsiloxane (PDMS) mold (25-µm channel width) was fabricated in a cleanroom using Photoresist SU-8 2025 (Kayaku Advanced Materials) following standard procedures, including spin coating, soft baking, laser exposure, post-exposure baking, development and hard baking. The mold thickness was measured using Zygo 3D Optical Profiler to be ~25 µm. The mold was placed in a plastic petri dish, and the PDMS mixture (part A: part B = 10:1, GE RTV) was poured in. The petri dish was placed into a vacuum chamber and degassed for ~30 minutes and then placed into a 70 °C oven and incubated for >2 hours or overnight. The cured PDMS slab was cut into a similar size as a 1 × 3-inch glass slide and stored at room temperature until use. The barcoding flow clamps and lysis clamps were fabricated through laser-cutting an acrylic plastic plate. After each DBiT-seq experiment, the PDMS chip can be reused by cleaning with 30-minute sonication in 1 M NaOH solution, 2 hours soaking in deionized water, 10-minute sonication in isopropanol and air dry at room temperature.

### Microscope setup

The tissue image and two flow channel/tissue images were scanned with the Invitrogen EVOS M7000 imaging system using a ×10 objective. Images were taken with mono-color mode and stitched with ‘More Overlap’ settings. The stitched images were saved into TIFF format and later aligned with spatial transcriptome and proteome data.

### DNA oligos and ADTs

DNA oligos used were all synthesized by Integrated DNA Technologies with high-performance liquid chromatography (HPLC) purification. All DNA oligos received were dissolved in RNase-free water at a 100 µM concentration and stored at −20 °C until use. All the DNA oligos used are listed in Supplementary Table 2. The barcode A and B oligos are listed in Supplementary Table 1. Barcode A contains three functional regions: a poly(T) region, a spatial barcode region and a ligation linker region. Poly(T) region hybrids with poly(A) tail of mRNA serve as the RT primer. The spatial barcode defines the row locations, and the ligation linker region was to be ligated with barcode B. Barcode B includes four functional regions: one ligation linker region, a spatial barcode region, a UMI region and a PCR primer region. The ligation linker region was to be ligated to barcode A. The spatial barcode region shows the column locations. Barcode B was also functionalized with 5′ biotin.

ADTs for membrane proteins were purchased from BioLegend and are listed in Supplementary Table 2. Three antibody cocktail products are 273 antibodies cocktail for humans with nine isotype control antibodies (cat. no. 99502) and 189 antibodies cocktail for mice with nine isotype control antibodies (cat. no. 99833).

### Tissue preparation

OCT embedded mouse spleen (mouse CD1 spleen frozen sections, MF-701), colon (mouse CD1 colon frozen sections, MF-311), intestine (CD1 intestine, jejunum frozen sections, MF-308) and kidney (mouse CD1 kidney frozen sections, MF-901) sections were purchased from Zyagen and stored at −80 °C until use. In a typical protocol, OCT tissue blocks were sectioned into 10-µm-thickness sections and placed in the center of poly-l-lysine slides (Electron Microscopy Sciences, 63478-AS) and shipped with dry ice. The human tonsil sections (human tonsil frozen sections, HF-707) were also purchased from Zyagen. Human skin samples were obtained from the Yale Department of Neurology and sectioned into a 10-µm thickness. For human skin sample, a 68-year-old male with a history of bullous pemphigoid in clinical remission, off systemic immunosuppressive or immunomodulatory therapy, was immunized for COVID-19 with the Moderna mRNA vaccine under FDA Emergency Use Authorization as standard of care; biopsies were performed on the immunized and unimmunized skin of the upper arms just below the vaccination site 2 days after the second and third vaccine doses. Informed consent was obtained from this patient. This study was approved by the institutional review board at Yale School of Medicine (protocol ID: 2000027055).

### Spatial-CITE-seq profiling of tissue

OCT embedded tissue sections stored in a −80 °C freezer were left on the working bench for 10 minutes. Sections were then fixed with 4% formaldehyde for 20 minutes and washed three times with 1× PBS with 0.05 U μl^−1^ RNAse Inhibitor (Enzymatics, 40 U μl^−1^). The tissue was then permeabilized with 0.5% Triton X-100 in 1× PBS for another 20 minutes before washing three times with 1× PBS. The sections were quickly dipped in RNase-free water and dried with air. We then covered the tissue using 1× blocking buffer with 0.05 U μl^−1^ RNAse Inhibitor (Enzymatics, 40 U μl^−1^) and incubated at 4 °C for 10 minutes. After washing three times with 1× PBS buffer, ADT cocktails (diluted 20 times from original stock) from BioLegend were added onto the tissue and incubated for 30 minutes at 4 °C. The ADT cocktail was removed by washing three times with 1× PBS, and the slide was dipped in water briefly to remove any remaining salts. A whole tissue image scan was performed with an EVOS microscope using a ×10 objective.

In-tissue reverse transcription was conducted by flowing reverse transcription reagents into each of the 50 channels. We prepared the reverse transcription mix by adding sequentially 50 μl of 5× RT buffer (Thermo Fisher Scientific), 7.8 μl of RNase-free water, 1.6 μl of RNAse Inhibitor (Enzymatics), 3.2 μl of SUPERase-In RNase Inhibitor (Ambion), 12.5 μl of 10 mM dNTPs each (Thermo Fisher Scientific), 25 μl of Maxima H Minus Reverse Transcriptase (Thermo Fisher Scientific) and 100 μl of 0.5× PBS-RI (0.5× PBS + 1% RNAse Inhibitor from Enzymatics) into a 1.5-ml tube (Extended Data Table 3). The mix was enough for a DBiT-seq chip with 50 channels and was further mixed with individual barcode A (25 μM in water) with a 4:1 volume ratio. The first PDMS chip was then placed on top of the tissue section, and customized plastic clamps were applied to the chip to seal tightly the PDMS chip with the tissue. The slide was imaged again with an EVOS microscope to record the locations of the channels. A total volume of 5 µl of reverse transcription mix and barcode A was loaded into each inlet well on the first PDMS chip. After loading and carefully removing air bubbles inside each well, a vacuum adapter made with acrylic plastic was placed on the outlet wells of the chip, and solutions were then vacuumed through the 50 channels. After 2 minutes, the vacuum was turned off, and the chip was placed into a wet box and incubated first at room temperature for 30 minutes and then for 90 minutes at 42 °C. When the RT reaction was completed, the channels were flushed with 1× NEB buffer 3.1 with 1% RNAse Inhibitor (Enzymatics) for 5 minutes. After removing the first PDMS chip, the tissue was dipped in RNase-free water and kept dry at 4 °C until the next step.

In-tissue ligation was performed in the second PDMS chip, which has 50 channels with orthogonal direction. The barcode B and ligation linker mix was first prepared by mixing barcode B (100 µM in water), 10 µl of ligation linker oligo (100 µM in water) and 20 µl of annealing buffer (10 mM Tris pH 7.5–8.0, 50 mM NaCl and 1 mM EDTA) in a PCR tube and then heated to 90–95 °C for 3–5 minutes before cooling to room temperature on the workbench. The mix was stored at 4 °C for short-term use or at −20 °C for long-term storage.

The ligation mix was prepared by adding into a 1.5-ml Eppendorf tube 68 µl of RNase-free water, 29 μl of 10× T4 ligase buffer (New England Biolabs (NEB)), 11 μl of T4 DNA ligase (400 U μl^−1^, NEB), 2 μl of RNAse Inhibitor (40 U μl^−1^, Enzymatics), 0.7 μl of SUPERase-In RNase Inhibitor (20 U μl^−1^, Ambion), 5.4 μl of 5% Triton X-100 and 116 µl of 1× NEB buffer 3.1 with 1% RNAse Inhibitor (40 U μl^−1^, Enzymatics). Then, 4 µl of ligation mix was mixed with 1 µl of barcode B (25 µM, with ligation linker) in a 96-well plate. The second PDMS chip was attached to the section and clumped together with an acrylic clump. The chip was scanned with the EVOS microscope to record the spatial locations of channels. Next, 5 µl of the above mixture was loaded into the inlet wells of the PDMS chip and vacuumed through each channel. The chip was transferred to a 37 °C oven and incubated for 30 minutes. The remaining solution in the inlets wells was removed, and wash buffer (1× PBS with 0.1% Triton X-100) was loaded and vacuumed through the channels continuously for 5 minutes. The PDMS chip was peeled off, and the tissue was dipped in water and dried with air.

The whole tissue section was digested by proteinase K to release the cDNAs. We prepare the lysis buffer by mixing 50 μl of 1× PBS, 50 μl of 2× lysis buffer (20 mM Tris pH 8.0, 400 mM NaCl, 100 mM EDTA and 4.4% SDS) and 10 μl of proteinase K solution (20 mg ml^−1^). A PDMS reservoir was placed on top of the region of interest, and the lysis mix was added. The reservoir was then clamped tightly with the slide to avoid any leakage and was sealed with parafilm. The tissue was lysed in a 55 °C oven for 2 hours, and the lysis was collected and kept in a −80 °C freezer until use.

cDNA extraction from the tissue lysate was performed in two steps. In the first step, all DNA was extracted from the lysate using the DNA purification kit (Zymo Research, ZD4014). We followed recommended protocols using a 5:1 ratio for the DNA binding buffer and lysate. In the second step, biotinylated cDNAs were captured with streptavidin beads (Dynabeads MyOne Streptavidin C1, Invitrogen). Before use, the beads were washed three times with 1× B&W buffer with 0.05% Tween 20 and dispersed into 100 µl of 2× B&W buffer. The beads were added into the purified cDNA with a 1:1 volume ratio and incubated with mild rotation at room temperature for 1 hour. Beads were cleaned twice with 1× B&W buffer and once using 1× Tris buffer with 0.1% Tween 20.

To add a second PCR handle to the cDNA strands, template switch was performed. We prepared the template switch reagents with standard protocol, using 44 µl of 5× RT buffer, 44 µl of Ficoll PM-400 solution, 22 µl of dNTPs, 5.5 µl of RNAse Inhibitor, 11 µl of Maxima H Minus Reverse Transcriptase, 5.5 µl of template switch oligo and 88 µl of water. The beads were resuspended into the mix, and the reaction was performed at room temperature for 30 minutes and then for 1.5 hours at 42 °C with rotation. After template switch, the beads were cleaned once with 1× PBST (0.1% Tween 20) and once with water.

We prepared the 220 µl of PCR mix with 110 µl of KAPA HiFi HotStart Master Mix, 8 µl of primer 1 (10 µM), 8 µl of primer 2 (10 µM), 0.5 µl of primer 2-citeseq (1 µM) and 91.9 µl of water. The cleaned Dynabeads were redispersed in this PCR mix, and the solution was split into four PCR tubes with 55 µl each. PCR was performed by first incubating at 95 °C for 3 minutes and then running 20 cycles at 98 °C for 20 seconds, 65 °C for 45 seconds and 72 °C for 3 minutes. To separate the cDNAs derived from RNA and cDNAs derived from ADT, we did the purification using 0.6× SPRI beads following standard protocol. Specifically, we added 120 µl of SPRI beads to 200 µl of PCR product solution and incubated for 5 minutes. The supernatant containing the ADT cDNAs was collected in a 1.5-ml Eppendorf tube. The remaining beads were cleaned with 85% ethanol for 0.5 minutes and then eluted with RNase-free water for 5 minutes. The cDNAs derived from mRNA were then quantified with Qubit and BioAnalyzer. For the supernatant, we added another 1.4× SPRI beads and incubated them for 10 minutes. The beads were cleaned once with 80% ethanol and redispersed in 50 µl of water. We did another 2× SPRI purification by adding 100 µl of SPRI beads and incubated for 10 minutes. After washing twice with 80% ethanol, we collected the cDNAs derived from ADTs by eluting them with 50 µl of RNase-free water.

The sequencing library of the two types of cDNA products was built separately. For cDNAs derived from mRNA, 1 ng of the cDNA was used, and the library was built using the Nextera XT Library Prep Kit (Illumina, FC-131-1024) using customized index strands and purified with 0.6× SPRI beads. For ADT cDNAs, the library was built with PCR. In a PCR tube, 45 µl of ADT cDNA solution, 50 µl of 2× KAPA HiFi PCR Master Mix, 2.5 µl of customized i7 index (10 µM) and 2.5 µl of P5 index (N501-citeseq, 10 µM) were mixed. PCR was performed at 95 °C for 3 minutes and then cycled at 95 °C for 20 seconds, 60 °C for 30 seconds and 72 °C for 20 seconds for a total of six cycles, and the reaction was finished with incubation at 72 °C for 5 minutes. The product was purified with 1.6× SPRI beads and then quantified with Qubit and BioAnalyzer. The libraries were sequenced with the NovaSeq 6000 system.

### scRNA-seq for human skin biopsy sample

Skin punch biopsies were placed immediately into MACS Tissue Storage Solution (Miltenyi Biotec, 130-100-008) and processed into single-cell suspensions using the Whole Skin Dissociation Kit (Miltenyi Biotec, 130-101-540) according to the manufacturer’s recommendations. In brief, the tissue was placed in the enzyme solution and incubated in a 37 °C water bath for 3 hours. Thereafter, the tissue cells were dissociated using the MACS Dissociator (Miltenyi Biotec, 130-093-235), pre-programmed for skin cell isolation (program h-skin-01). The cells were then resuspended in DMEM, and mononuclear cells were isolated by Ficoll-Paque PLUS (GE Healthcare) gradient centrifugation. Single-cell preparations were loaded into the Chromium Controller (10x Genomics) for emulsion generation, and libraries were prepared using the Chromium Single Cell 5′ Reagent Kit for version 1.1 chemistry per the manufacturer’s protocol. Libraries were sequenced on the NovaSeq 6000 for gene expression and BCR/TCR libraries.

### Data pre-processing

For cDNAs derived from mRNAs, the raw FASTQ file of Read 2 containing the UMI and barcode A and barcode B regions was first reformatted into the standard input format required by ST Pipeline version 1.7.2 (ref. ^23^) using customized Python script. Using recommended ST Pipeline parameters, the Read 1 was STAR mapped to either the mouse genome (GRCm38) or the human genome (GRCh38). The gene expression matrix contains the spatial locations (barcode A × barcode B) of the genes and gene expression levels.

For cDNAs derived from ADTs, the raw FASTQ file of Read 2 was reformatted the same way as cDNAs from RNA. Using default settings of CITE-seq-Count 1.4.2 (ref. ^24^), we counted the ADT UMI numbers for each antibody in each spatial location. The protein expression matrix contains the spatial locations (barcode A × barcode B) of the proteins and protein expression levels.

### Clustering and visualization

The clusters of RNA and protein expression matrix was generated using Seurat version 3.2 (ref. ^25^). The transcriptome data were normalized using the ‘SCTransform’ function. Normalized data were then clustered and UMAP was built with the dimensions set to 30, and cluster resolution was set to 0.5. Protein data were normalized using the centered log ratio (CLR) transformation method in Seurat version 3.2. All heat maps were plotted using ggplot2. Weighted nearest neighbor analysis were conducted using Seurat version 3.2 following default settings.

### scRNA-seq and spatial data integration

The cell types of skin biopsy section were annotated through integration analysis using the matched scRNA-seq data as the reference. The two datasets were normalized with the ‘SCTransform’ function in Seurat version 3.2 and then integrated into one dataset. After clustering, the spatial pixel data conformed well with the scRNA-seq data, and, thus, the cell types were assigned based on the scRNA-seq cell type annotation for each cluster (if two cell types presented in one cluster, the major cell types were assigned). SPOTlight was used to deconvolve the spatial spots^18^.

### Fluorescent staining of human tonsil

The CODEX imaging with six protein markers—CD21, CD31, CD3, CD90, CD279 and CD19—was conducted following standard PhenoCycler protocols with default settings. Highly multiplexed immunofluorescence imaging on a separate formalin-fixed, paraffin-embedded human tonsil tissue section was performed by sequential immunofluorescence staining on COMET using the FFeX technology previously described by Lunaphore Technologies^22,26^.

### Spatial-CITE-seq comparison with scCITE-seq

The scCITE-seq dataset was obtained from a published study^15^. It was first cleaned by removing cells with fewer than ten total ADT UMIs and further randomly downsampled to 10,000 cells. scCITE-seq and spatial-CITE-seq datasets were combined, normalized with ‘SCTransform’ in Seurat version 3.2 and then integrated into a single dataset to perform clustering analysis.

### Reporting summary

Further information on research design is available in the Nature Portfolio Reporting Summary linked to this article.

## Online content

Any methods, additional references, Nature Portfolio reporting summaries, source data, extended data, supplementary information, acknowledgements, peer review information; details of author contributions and competing interests; and statements of data and code availability are available at 10.1038/s41587-023-01676-0.

### Supplementary information

Reporting Summary

Supplementary Table 1 DNA barcodes used in this study.

Supplementary Table 2 Antibodies used in this study.

## Figures and Tables

**Extended Data Table 1:**
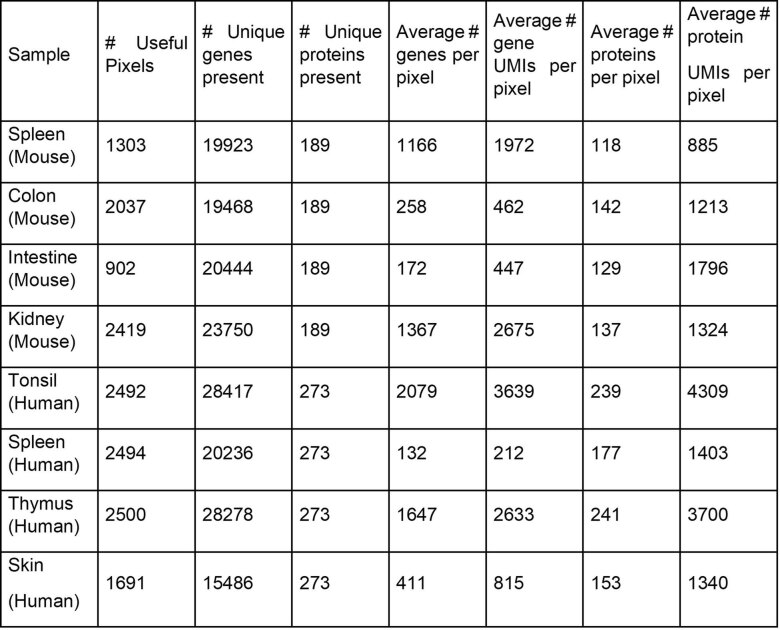
Summary of gene and protein counts for all the samples sequenced

**Extended Data Table 2:**
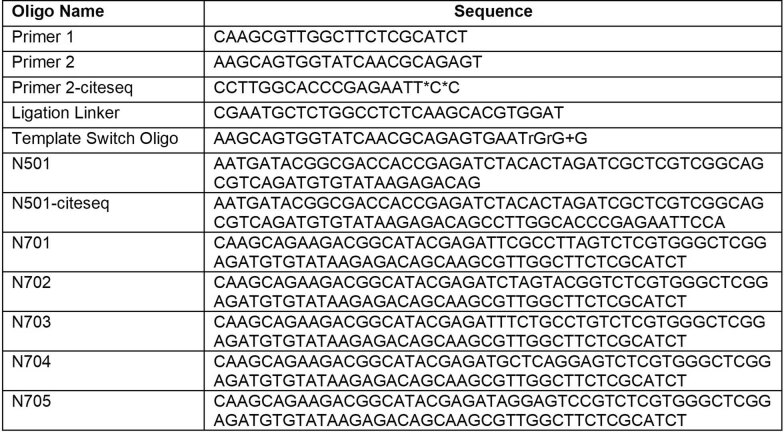
DNA oligos for PCR, ligation and library preparation

**Extended Data Table 3:**
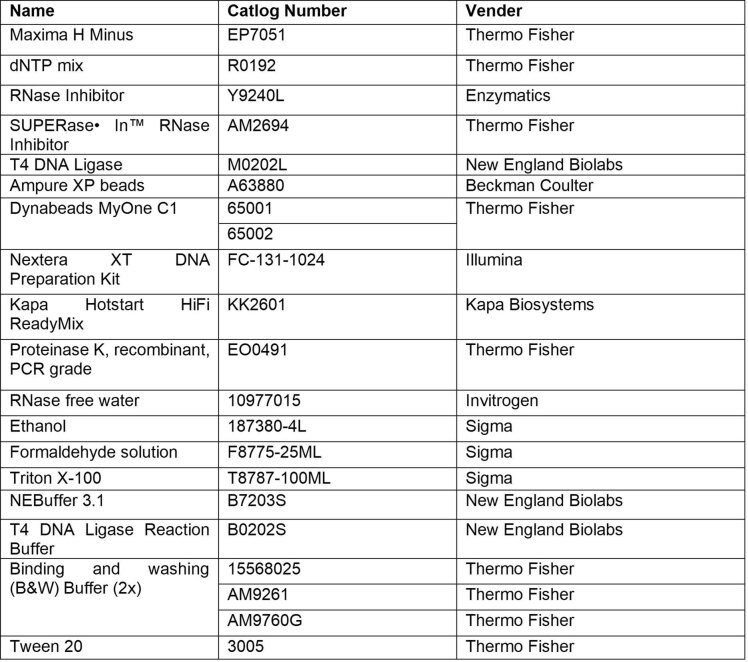
Chemicals and reagents used

## Data Availability

The sequencing data reported in this paper are available at the Gene Expression Omnibus (GSE213264). The high-resolution microscope images are available at 10.6084/m9.figshare.20723680.

## Code availability

The main R scripts used in this paper are available on GitHub: https://github.com/edicliuyang/Hiplex_proteome.
