# Supplementary material for: High-plex protein and whole transcriptome co-mapping at cellular resolution with spatial CITE-seq
Source: Nat Biotechnol. 2023 Feb 23;41(10):1405–9. doi: 10.1038/s41587-023-01676-0 (PMC10567548; doi:10.1038/s41587-023-01676-0)
Supplement: Supplementary file 1 — Reporting Summary [file 41587_2023_1676_MOESM1_ESM.pdf]

nature research

Corresponding author(s): Rong Fan

Last updated by author(s): 1/6/2022

Reporting Summary

Nature Research wishes to improve the reproducibility of the work that we publish. This form provides structure for consistency and transparency in reporting. For further information on Nature Research policies, see our Editorial Policies and the Editorial Policy Checklist.

Statistics

For all statistical analyses, confirm that the following items are present in the figure legend, table legend, main text, or Methods section.

n/a	Confirmed	
☐	☒	The exact sample size (n) for each experimental group/condition, given as a discrete number and unit of measurement
☒	☐	A statement on whether measurements were taken from distinct samples or whether the same sample was measured repeatedly
☒	☐	The statistical test(s) used AND whether they are one- or two-sided<br>*Only common tests should be described solely by name; describe more complex techniques in the Methods section.*
☒	☐	A description of all covariates tested
☒	☐	A description of any assumptions or corrections, such as tests of normality and adjustment for multiple comparisons
☐	☒	A full description of the statistical parameters including central tendency (e.g. means) or other basic estimates (e.g. regression coefficient) AND variation (e.g. standard deviation) or associated estimates of uncertainty (e.g. confidence intervals)
☒	☐	For null hypothesis testing, the test statistic (e.g. F, t, r) with confidence intervals, effect sizes, degrees of freedom and P value noted<br>*Give P values as exact values whenever suitable.*
☒	☐	For Bayesian analysis, information on the choice of priors and Markov chain Monte Carlo settings
☒	☐	For hierarchical and complex designs, identification of the appropriate level for tests and full reporting of outcomes
☒	☐	Estimates of effect sizes (e.g. Cohen's d, Pearson's r), indicating how they were calculated

Our web collection on statistics for biologists contains articles on many of the points above.

Software and code

Policy information about availability of computer code

Data collection	Seurat V3.2, ST Pipeline v1.7.2 , The PhenoCycler System, CITE-seq-Count 1.4.2
Data analysis	The main R (Version 4.2.1) scripts used in this paper was deposited to Github: https://github.com/edicliuyang/Hiplex_proteome.

For manuscripts utilizing custom algorithms or software that are central to the research but not yet described in published literature, software must be made available to editors and reviewers. We strongly encourage code deposition in a community repository (e.g. GitHub). See the Nature Research guidelines for submitting code & software for further information.

Data

Policy information about availability of data

All manuscripts must include a data availability statement. This statement should provide the following information, where applicable:

- Accession codes, unique identifiers, or web links for publicly available datasets
- A list of figures that have associated raw data
- A description of any restrictions on data availability

The sequencing data reported in this paper are available at GEO: GSE213264. The high-resolution microscope images were uploaded to https://doi.org/10.6084/m9.figshare.20723680

Field-specific reporting

Please select the one below that is the best fit for your research. If you are not sure, read the appropriate sections before making your selection.

☒ Life sciences ☐ Behavioural & social sciences ☐ Ecological, evolutionary & environmental sciences

For a reference copy of the document with all sections, see nature.com/documents/nr-reporting-summary-flat.pdf

Life sciences study design

All studies must disclose on these points even when the disclosure is negative.

Sample size: The study mainly focused on demonstrating a new spatial technique; the best way to demonstrate it is to test various tissue types. The sample size was thus not determined in this study.

Data exclusions: No data was excluded from the analyses.

Replication: There are no replicates in this study, mainly due to the fact there are enough data collected, which can support the feasibility of the current technique.

Randomization: There are no defined sample groups in this research, so randomization is not relevant to this study.

Blinding: There are no defined sample groups in this research, so Blinding is not relevant to this study.

Reporting for specific materials, systems and methods

We require information from authors about some types of materials, experimental systems and methods used in many studies. Here, indicate whether each material, system or method listed is relevant to your study. If you are not sure if a list item applies to your research, read the appropriate section before selecting a response.

Materials & experimental systems

n/a	Involved in the study	
☐	☒	Antibodies
☒	☐	Eukaryotic cell lines
☒	☐	Palaeontology and archaeology
☒	☐	Animals and other organisms
☐	☒	Human research participants
☒	☐	Clinical data
☒	☐	Dual use research of concern

Methods

n/a	Involved in the study	
☒	☐	ChIP-seq
☒	☐	Flow cytometry
☒	☐	MRI-based neuroimaging

Antibodies

Antibodies used: Human antibody cocktail, Biolegend, Cat No. 99502; Mouse antibody cocktail, Biolegend, Cat No. 99833

Validation: These two antibody cocktails are pre-titrated, lyophilized TotalSeq™ panels.

Human research participants

Policy information about studies involving human research participants

Population characteristics: A 68-year-old male with a history of bullous pemphigoid in clinical remission, off systemic immunosuppressive or immunomodulatory therapy, was immunized for COVID-19 with the Moderna mRNA vaccine under FDA EUA as standard of care. Biopsies were performed on the immunized and unimmunized skin of the upper arms just below the vaccination site 2 days post the second and third vaccine doses.

Recruitment: Recruitment was performed through public announcements and through oral dissemination within the research arena and in the dermatology clinic setting which could result is self selection bias for individuals highly motivated to participate in research. This bias could result in participants with a disease background, as in our subject with autoimmune skin disease in remission off therapy. The participant was informed of potential risks and provided written and oral consent prior to participation.

Ethics oversight: This study was approved by the Institutional Review Board at the Yale School of Medicine (Protocol ID#; 2000027055).

Note that full information on the approval of the study protocol must also be provided in the manuscript.
